# Understanding psychological distress among mothers in rural Nepal: a qualitative grounded theory exploration

**DOI:** 10.1186/1471-244X-14-60

**Published:** 2014-03-01

**Authors:** Kelly Clarke, Naomi Saville, Bishnu Bhandari, Kalpana Giri, Mamita Ghising, Meena Jha, Sonali Jha, Jananee Magar, Rinku Roy, Bhim Shrestha, Bhawana Thakur, Rinku Tiwari, Anthony Costello, Dharma Manandhar, Michael King, David Osrin, Audrey Prost

**Affiliations:** 1 University College London Institute for Global Health, 30 Guilford Street, London WC1N 1EH, UK; 2 Mother and Infant Research Activities (MIRA), Thapathali, Kathmandu, Nepal; 3 St Albans, Hertfordshire, UK; 4 Transcultural Psychosocial Organisation Nepal, Baluwatar, Kathmandu, Nepal; 5 Research Department of Mental Health Sciences, University College London, Charles Bell House, 2nd Floor, 67-73 Riding House Street, London W1W 7EJ, UK

**Keywords:** Nepal, South Asia, Psychological distress, Postnatal depression, Perinatal common mental disorders, Maternal mental health, Rural health

## Abstract

**Background:**

There is a large burden of psychological distress in low and middle-income countries, and culturally relevant interventions must be developed to address it. This requires an understanding of how distress is experienced. We conducted a qualitative grounded theory study to understand how mothers experience and manage distress in Dhanusha, a low-resource setting in rural Nepal. We also explored how distressed mothers interact with their families and the wider community.

**Methods:**

Participants were identified during a cluster-randomised controlled trial in which mothers were screened for psychological distress using the 12-item General Health Questionnaire (GHQ-12). We conducted 22 semi-structured interviews with distressed mothers (GHQ-12 score ≥5) and one with a traditional healer (*dhami*), as well as 12 focus group discussions with community members. Data were analysed using grounded theory methods and a model was developed to explain psychological distress in this setting.

**Results:**

We found that distress was termed *tension* by participants and mainly described in terms of physical symptoms. Key perceived causes of distress were poor health, lack of sons, and fertility problems. *Tension* developed in a context of limited autonomy for women and perceived duty towards the family. Distressed mothers discussed several strategies to alleviate tension, including seeking treatment for perceived physical health problems and *tension* from doctors or *dhamis*, having repeated pregnancies until a son was delivered, manipulating social circumstances in the household, and deciding to accept their fate. Their ability to implement these strategies depended on whether they were able to negotiate with their in-laws or husbands for resources.

**Conclusions:**

Vulnerability, as a consequence of gender and social disadvantage, manifests as psychological distress among mothers in Dhanusha. Screening tools incorporating physical symptoms of *tension* should be envisaged, along with interventions to address gender inequity, support marital relationships, and improve access to perinatal healthcare.

## Background

Psychological distress, which includes depressive, anxiety, panic and somatic disorders, is a major cause of disability among pregnant and postnatal women. Rates are highest in low and lower middle-income countries, where distress affects 16% (95% CI 15.4-15.9) of women during pregnancy and 20% (19.5-20.0) in the postnatal period [1]. The way in which distress is experienced and expressed varies across cultures, and this has considerable implications for understanding and treating it [2]. For example, qualitative studies in South Asia have shown that, while distressed pregnant and postnatal women experience symptoms of depressive biomedical disorders, they interpret their symptoms as social constructs related to economic difficulties, poor marital relations, and having too many daughters [3,4]. Help-seeking behaviours among these women are diverse and include seeking medical treatment for somatic symptoms and reproductive health complaints, faith healing, and developing strategies to alleviate poverty and resolve family problems [3-5].

There is evidence of a high burden of maternal mental illness in Nepal: estimates of distress in the postnatal period range from 5 to 12%, and suicide is the leading cause of death among women of reproductive age [6-9]. Quantitative studies have shown that poor reproductive health, son preference, and socioeconomic disadvantage are important predictors of distress among Nepalese mothers [10,11]. To date however, there have been no qualitative studies of perinatal distress in Nepal to contextualise findings from these quantitative studies and to guide intervention development.

We conducted a qualitative study of perinatal psychological distress in Dhanusha district, in the plains region of southern Nepal. We developed a grounded theory model to understand community perceptions and mothers’ experiences of distress, explore distressed mothers’ interactions with family members and the wider community, and identify strategies used by mothers to deal with distress.

## Methods

### Setting

Participants were identified during a cluster-randomised controlled trial (cRCT) conducted in Dhanusha (trial registration ISRCTN87820538). The trial evaluated the impact of participatory women’s groups on neonatal mortality and several secondary outcomes [12]. One of these outcomes was postnatal psychological distress, measured using the 12-item General Health Questionnaire (GHQ-12), which has been validated in Nepal [13]. Dhanusha has a population of 754,777, and comprises 102 administrative units called Village Development Committees (VDCs). Most people are Hindu (89%), though there is a substantial Muslim population [14]. The four most populous caste/ethnic groups are: Yadav (18%), Muslim (9%), Kewat (6%) and Teli (5%) [15]. At 51% (61% males and 40% females), the literacy rate in Dhanusha is lower than the national average of 66% [14]. Maithili is the most widely spoken language, although there are also Nepali-speaking (*Pahadi*) communities. People commonly live in extended families and married women live with their husbands’ families.

Child and maternal mortality rates are high: in control clusters the neonatal mortality rate was 35 per 1000 livebirths and the maternal mortality ratio was 223 per 100,000 livebirths during the trial (2006-11). There is one public zonal hospital in Janakpur (the district municipality) to serve Dhanusha and five other districts, and five primary healthcare centres, nine health posts and 88 sub-health posts, although people commonly consult with private practitioners. Public mental health services in Nepal are concentrated in large urban centres. There are no public mental health facilities in Dhanusha, although one NGO was funding a monthly mental health clinic in Janakpur during the study.

### Participants and data collection

Data comprised transcripts from 12 focus group discussions (FGDs) and 23 semi-structured interviews, and field notes. Although we selected a grounded theory approach from the outset of the study, we were unable to carry out theoretical sampling due to time and financial constraints. Interview and FGD participants were therefore purposively sampled prior to data collection [16].

### Focus group discussions

We conducted FGDs to explore community perceptions and experiences of perinatal distress. Local women’s groups, which were supported by facilitators in undertaking a participatory learning and action intervention during the Dhanusha cRCT, presented a strategic way to engage with women in the local communities. We recruited members and facilitators of six women’s groups out of a total of 270. Facilitators were female community health volunteers or local women elected by group members. Although groups targeted women of reproductive age, group members also included older and unmarried women. Only mothers who delivered during the study period were eligible to participate in the Dhanusha cRCT [12].

In order to elicit a broad range of perspectives on distress we sampled women’s groups in six VDCs with diverse populations. We purposively sampled those in Nepali and Maithili-speaking VDCs. Compared to their Nepali-speaking neighbours, Maithili-speaking communities tend to be poorer, less educated, and women have more restricted roles. We anticipated higher levels of distress in poorer VDCs and in those where women have less autonomy. Sampling VDCs based on language enabled us to take this into account. VDCs were also selected to represent caste groups and religions, as well as varying levels of remoteness from Janakpur. We conducted four additional FGDs with non-group members in control VDCs because FGDs in intervention VDCs may have overemphasised issues linked to ongoing women’s group activities, including nutrition and perinatal and infant health. In these FGDs we purposively sampled younger (<30 years) Maithili-speaking women who were under-represented in FGDs in intervention VDCs. Characteristics of VDCs purposively sampled for the FGDs are presented in Table 1.

**Table 1:** Characteristics of Village Development Committees in which focus group discussions were held

**Village Development Committee**	**Women’s groups intervention cluster**	**NMR (Aug 2006-Jun 2009)**	**Percentage Muslim**	**Percentage Madheshi (Maithilli-speaking) ethnicity**	**Percentage Dalit**	**Distance from Janakpur municipality and the zonal hospital**
Mukhiyapatti	Yes	24.3	10.5	95.3	11.8	Difficult access by road which is prone to flooding
Phulgama	Yes	37.2	1.9	95.7	19.8	Easy access
Basaiya	No	26.1	17.5	91. 4	31.1	Easy access, borders Janakpur
Lohana	Yes	18.1	52.3	95.0	20.4	Easy access as it borders the municipality
Sakuwa Mahendranagar	No	30.9	15.5	90.3	24.3	Easy road access by bus
Mansingpatti	No	37.2	0.3	97.5	26.3	Easy access
Dhalkebar	Yes	37.8	0.4	78.6	10.4	Easy road access by bus
Sapahi	No	42.8	13.2	97.8	19.3	Easy road access by bus
Bharatpur	Yes	43.5	14.5	71.8	23.4	Easy road access by bus but far
Thadi Jhija	Yes	61.6	31.1	92.2	40.7	Far but has train access

Notes: data are taken from the Dhanusha cRCT database; NMR, neonatal mortality rate per 1000 livebirths.

We designed FGD topic guides with locally adapted vignettes of postnatal depression to elicit participants’ explanatory models of psychological distress. The guides featured open questions about the illness experience, including: ‘What causes the illness?’ ‘What is the illness?’ ‘What should be done to address the illness?’ ‘How will the illness turn out?’ [17]. Topic guides were translated from English into Nepali and Maithili.

### Interviews

We conducted semi-structured interviews to explore experiences of distressed mothers in the perinatal period, and strategies to deal with distress. We purposively identified participants from a total of 1272 mothers who had participated in the Dhanusha cRCT and completed the GHQ-12 in the previous two months. We identified participants with a GHQ-12 score ≥5 as some studies have found that this threshold discriminates well for psychological distress among mothers [18-20]. Furthermore, enough mothers scored above this threshold to enable us to purposively sample based on severity of distress and demographic factors. Out of 1272 mothers, 116 had a GHQ-12 score ≥5. We included all mothers with GHQ-12 scores ≥8 in order to oversample those with high levels of distress. We grouped the rest according to caste and ethnicity, and preferentially included Dalit and Muslim mothers because these groups had the highest mean GHQ-12 scores in preliminary analyses of the Dhanusha cRCT data. We also included a mother from the Yadav group since this is the most populous community in Dhanusha. Four mothers from the purposively selected sample were unavailable for interview: one refused and three were staying outside the district.

The interview topic guide comprised open questions about emotional experiences, factors that contributed to happiness or unhappiness, help-seeking behaviours, and help that was or would have been useful during pregnancy and postnatally. The topic guide was developed through discussions and role-play with the data collection team in order to identify a suitable interview structure and locally appropriate terms. To enquire about feelings, we asked, ‘what thoughts were playing in your mind?’ (Maithili: *Aahanke mon me kon tarahake vichar sab abait chhalaik?* Nepali: *Tapai ko man ma kun kora kheli raheko thiyo?*), and ‘did you have any bad or negative thoughts?’ (Maithili: *Aahanke mon me kono kharab athave nahi nik vichar abait chhalaik?* Nepali: *Ke tapaai ko man ma kunai naramro athava kuvichar aaunthyo?*). The topic guide was written in Maithili and translated into English.

We also conducted an interview with a traditional healer (*dhami*) because many participants had consulted with these practitioners. The healer was selected because he was well known in Dhanusha for treating women with fertility problems. We used the FGD topic guide to elicit his views on perinatal psychological distress, as well as his personal experience of treating mental illness.

The Dhanusha cRCT was managed by staff at MIRA (Mother and Infant Research Activities). Female MIRA staff experienced in working with women’s groups facilitated the FGDs and conducted the semi-structured interviews. The first author attended the majority of FGDs and interviews. Participants were not reimbursed for taking part in interviews or FGDs.

### Data analysis

**Figure 1:**
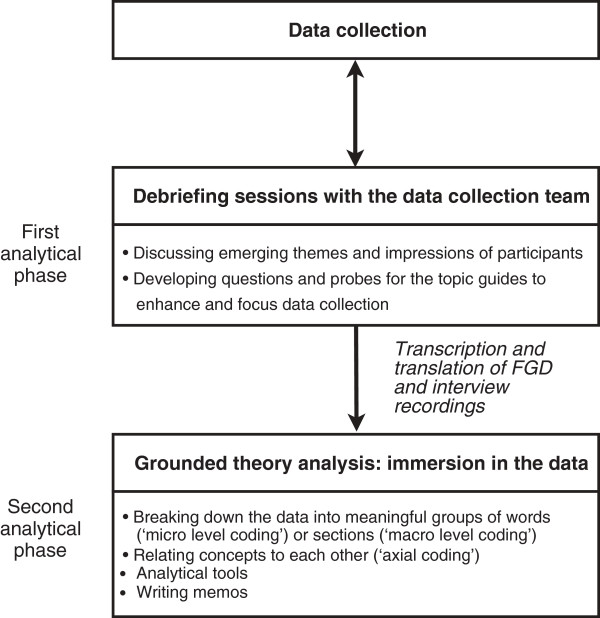
**Two-stage qualitative analysis procedure.** Flow chart showing the two-stage procedure used to analyse the data, comprising debriefing sessions with the data collection team after each interview and FGD, and a grounded theory analysis.

Audio recordings of FGDs and interviews were transcribed in Maithili or Nepali and translated into English. Figure 1 shows our two-stage analysis procedure, comprising debriefing sessions with the data collection team after each interview and FGD, and a grounded theory analysis following the Straussian approach [16].

We used coding techniques to break down data into meaningful groups of words or larger sections of the transcript that were labelled with concepts or categories. To increase rigor of analysis, KC and AP coded a transcript independently and compared and discussed their coding schemes. We used axial coding to relate concepts to each other, and analytical tools including the ‘flip-flop’ technique of looking at the effect of inserting the opposite meaning of a statement, and making comparisons between incidents, events and actions [16]. Diagrams and memos were used to document use of analytical tools and develop properties and dimensions of emerging concepts and categories. We used Nvivo 10 software for coding and writing memos [21].

Theoretical sampling – using the emerging theory to direct data collection – was not possible because data were collected prior to the grounded theory analysis. However, we adhered to the principles of theoretical sampling by revising questions and probes in the topic guide based on discussions during debriefing sessions, using emerging concepts to inform the order in which transcripts were analysed (enabling us to define properties of concepts as they were delineated and focus the analysis), revisiting incidents in previously analysed data whose significance only became apparent in later stages of the analysis [16].

We used the Paradigm Model to identify context, defined as a set of conditions in which problems or situations arise that provoke action, interaction and emotion (‘process’) [22]. We identified a core category according to the following criteria: it should be related to all other categories, appear frequently in the data, be logical and consistent with the data, be abstract enough that it can be used in other research areas, and have increasing explanatory power as additional categories are related to it [16]. We achieved theoretical integration of categories by reviewing and sorting through memos and diagrams, and summarising what the data communicated [16]. The validity of the final model was discussed among the authors and revised accordingly.

### Ethical considerations

The Dhanusha cRCT had ethical clearance from the Nepal Health Research Council and the Ethics Committee of the Institute of Child Health and Great Ormond Street Hospital. We obtained further ethical approval for the qualitative study from University College London’s Research Ethics Committee (application number 2656/001). We sought informed verbal consent from all FGD and interview participants and provided information about a mental health clinic in Janakpur. We arranged an advance appointment for one mother deemed to be too distressed to wait until the next clinic.

## Results

FGD and interview participant sampling frames are shown in Tables 2 and 3. We conducted FGDs with 105 local women, women’s group members, and group facilitators. FGD participants were Hindu or Muslim women between the ages of 18 to 73 years. Most of the group members and local women had children but received no education. Fifteen of the facilitators and co-facilitators had received some education. We interviewed 22 mothers with GHQ-12 scores ≥5, as well as one traditional healer. The average age of mothers interviewed was 25 years (standard deviation = 4). Twelve mothers were Hindu and ten were Muslim. Mothers had between one and four children and all but one received no education. We sampled mothers from both poorer and richer households. We use pseudonyms, selected to reflect ethnicity and caste, to preserve participant anonymity.

**Table 2:** Characteristics of focus group discussion participants

**VDC location of the FGD**	**Number of participants**	**Age range**	**Education**				**Religion**			**No. of children**			
			**None**	**Class 1-6**	**Class 7-12**	**SLC pass**	**Hindu**	**Muslim**	**Buddhist**	**0**	**1-3**	**4-6**	**>6**
***FGDs with women’s group members***													
Dhalkebar	10	18-70	7	1	1	1	10	0	0	1	5	3	0
Mukhiyapatti	9	25-50	9	0	0	0	9	0	0	0	2	7	0
Lohana	9	32-60	9	0	0	0	0	9	0	0	2	4	3
Phulgama	12	25-60	2	4	5	0	12	0	0	-	-	-	-
Bharatpur	12	19-73	5	2	4	1	0	0	12	2	9	0	1
Thadi Jhija	10	19-44	7	1	2	0	10	0	0	0	6	4	0
***FGDs with women’s group facilitators and co-facilitators***													
Dhalkebar	7	23-60	1	2	4	0	7	0		0	6	1	0
Phulgama	12	20-60	3	4	5	0	12	0	0	-	-	-	-
***FGDs with local women in control VDCs***													
Basaiya	6	25-40	6	0	0	0	6	0	0	0	5	1	0
Mansinghpatti	6	24-28	2	1	1	2	6	0	0	0	6	0	0
Sakuwa Mahendranagar	6	19-45	3	3	3	0	6	0	0	0	6	0	0
Sapahi	6	22-45	5	0	1	0	6	0	0	0	5	1	0

- : data were not collected.

**Table 3:** Characteristics of interview participants

**Pseudonym**	**VDC name**	**Religion**	**Caste**	**GHQ-12 score**	**Parity**	**No. of sons**	**Age**	**Education**	**Asset quintile**
Saikala Khatun	Suga Madhukari	Muslim	Khatun	10	3	≥1	32	None	Next richest
Rina Devi	Khajuri Channa	Hindu	Dalit	8	3	0	30	None	-
Lalita Devi	Ekhari	Hindu	Sudi/Teli	5	2	0	-	None	Next richest
Babita Devi	Bindhi	Hindu	Mandal	6	2	1	27	None	Next richest
Hajara Khatun	Lohana	Muslim	Sheikh	7	3	-	25	None	Second poorest
Pramila Devi	Mansingpatti	Hindu	Dalit	11	1	0	25	None	-
Gita Devi	Bindhi	Hindu	Sudi/Teli	5	1	1	19	None	Richest
Khabira Khatun	Bindhi	Muslim	Muslim	6	1	1	21	None	Richest
Roshana Khatun	Kanakpatti	Muslim	Ansari	6	4	3	-	-	-
Samina Khatun	Lohana	Muslim	Sheikh	6	2	1	-	-	-
Naima Khatun	Bindhi	Muslim	Sudi/Teli	5	4	≥1	26	None	Richest
Sunita Devi	Mansingpatti	Hindu	Dalit	9	2	2	25	None	Richest
Sagira Khatun	Lohana	Muslim	Sheikh	8	3	≥1	25	None	Middle
Amala Devi	Bindhi	Hindu	Mandal	5	3	1	24	None	Poorest
Sarita Devi	Khajuri Channa	Hindu	Dalit	7	-	0	-	-	-
Punita Devi	Lohana	Hindu	Mandal	9	1	1	18	None	Middle
Sahida Khatun	Bindhi	Muslim	Raine	5	3	2	25	Muslim school	Richest
Sanjita Devi	Devdiya	Hindu	Dalit	8	3	1	27	None	Poorest
Sulekha Khatun	Bindhi	Muslim	Dhobi	5	2	0	26	None	Next richest
Somani Devi	Lagma Gathaguthi	Hindu	Dalit	5	4	0	35	None	Middle
Radha Devi	Devdiya	Hindu	Dalit	8	2	0	23	None	Middle
Rajina Khatun	Kanakpatti	Muslim	Ansari	8	2	≥1	22	None	Poorest

- : data unknown; asset quintiles were calculated through principle components analysis of data from the Dhanusha cRCT.

### Grounded theory model for psychological distress

**Figure 2:**
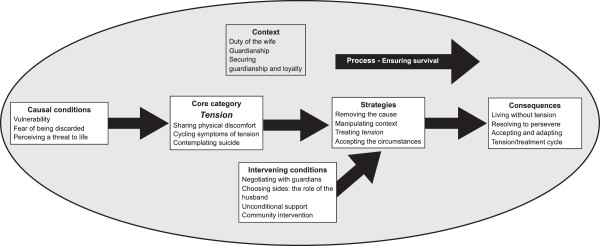
**Qualitative model of psychological distress among mothers in Dhanusha, Nepal.** Grounded theory model identifying the context, causal conditions, core category, process, intervening conditions, strategies and consequences associated with psychological distress among mothers in Dhanusha, Nepal.

The model for psychological distress among mothers in Dhanusha is presented in Figure 2. This section describes each component of the model.

### Context in which distress developed

Participants, including distressed mothers, non-distressed local women, group members and facilitators, provided detailed information on the context in which distress arose. Many described a concept that framed women’s lives and their relationship to their families: the ‘duty of the wife’ (*patni ke kartavya* in Maithili; *swaasni maanchhe ko kartavaya* in Nepali), comprised domestic tasks such as cooking for the family, cleaning, washing clothes, caring for children and looking after animals, but also reproductive obligations (bearing children, especially sons) and traditional or spiritual responsibilities, including a period of confinement in the first six days after delivery:

*Women are kept in the postpartum room after delivery. They do not make a single hole for air. They don’t allow her to change her clothes. The room is filled with smoke. She is given separate drinking water because they think she is untouchable. They don’t give her proper food to eat - they give ginger, raw sugar and halwa.* (Manju Devi, facilitator, Phulgama)

Guardians (parents in-law) were responsible for defining the duties of wives and providing daughters in-law with ‘protection’ and ‘management’, collectively referred to as ‘guardianship’. Guardians made decisions about their daughter-in-law’s healthcare and diet, and sometimes stipulated how many children she should have. In all FGDs, participants described quarrels and violence between guardians and daughters-in-law. When guardianship was withdrawn, distressed mothers were forced to return to their parents’ home (*nahira* in Maithili; *maiti* in Nepali), where they were taunted and “blamed” by neighbours who felt that married women should live with their husbands. Guardianship was portrayed as essential for survival and social acceptance, and a local woman taking part in an FGD described how a widow living in her community without guardians was unable to feed and clothe her family. Distressed mothers seemed to fulfil their wifely duties to secure guardianship, be perceived as good wives, make their husbands happy and maintain family ‘honour*’* (*Izzat* in Maithili and Nepali):

*I haven’t done any foolish things, like consuming poison, or hanging myself, despite facing so many crises in life. No one could say anything about my action in my parents’ home or in- laws’. I do feel like running away with someone but I haven’t to keep my parents’ and in-law’s honour.* (Rina Devi, mother, aged 30, Dalit caste, GHQ-12 score of 8, Khajuri Channa VDC)

### Core category: *tension*

Both distressed mothers and FGD participants used the English word *tension* to refer to a state of psychological distress, as well as a stressful event or series of events. They described having varying amounts of *tension*, and also relentless *tension* (getting rid of one *tension* but another appearing shortly after). *Tension* was described as ‘having many thoughts playing in your mind’ (*aahaanke monme bahut tarahke baat abait chaik* in Maithili; *tapaaiko manma dherai kuraharu kheli raheka chhan* in Nepali), and being distracted, worried (*chintit* in Maithili and Nepali), despairing (“how will I survive?”) and unable to complete housework. Local women and group members or facilitators used *tension* to refer to their own distress, and distress experienced by individuals they perceived to be enduring hardship, whereas ‘mad’ (*bataah* in Maithili; *baulaahaa* in Nepali) was used for individuals behaving erratically without an apparent cause. More educated Nepali-speaking group members used the term ‘mentally disturbed’ (*mansik roopsa aswasth* in Maithili; *mansik rup le aswasth* in Nepali). Local non-group members described how a mother with symptoms of distress would be labelled as a ‘witch’ (*daain* in Maithili; *bokshi* in Nepali) in their community.

Many of the symptoms of *tension* were common to International Classification of Diseases (ICD-10) diagnoses of depression and Generalised Anxiety Disorder (GAD), though loss of interest or pleasure, self-blame, and low self-confidence were not mentioned (Table 4). Reporting of physical symptoms (lack of energy, disturbed sleep, loss of appetite, and aches and pains) took precedence over affective symptoms (feeling sad, anxious or irritable). They were emphasised, repeated several times, and described in detail. Distressed mothers commonly communicated the severity of physical symptoms by stating how their weakness, tiredness or dizziness disrupted daily activities such as eating, sleeping and completing housework. They rarely described their emotions, despite being asked several times during an interview, and said that they shared physical but not emotional problems with family members since the latter would cause upset.

**Table 4:** **Quotes from participants describing symptoms of depression, generalised anxiety and *tension***

**Symptoms**	**Supportive quotes from participants**	**Participant’s GHQ-12 score**	**Participant’s caste**
***ICD-10 symptoms of depression***			
Persistent sadness	“I felt sad and my heart was also not stable.”	n/a	Yadav
	“When I became sad I felt weak and kept thinking about these things.”	9	Mandal
Fatigue or low energy	“I was so weak, didn’t feel like doing anything.”	7	Muslim
	“I was too weak to carry a bucket full of water, so I brought half a bucket of water to do some housework.”	7	Muslim
Disturbed sleep (also GAD)	“Even now I can’t sleep if I get worried.”	8	Dalit
	“I was feeling sleepy all the time because of my worry.”	8	Muslim
Poor concentration or indecisiveness (also GAD)	“I couldn’t understand the work because of *tensions*.”	7	Muslim
Poor or increased appetite	“I lost my appetite.”	11	Dalit
	“I have so much *tension*. I have become weak because I don’t eat properly.”	5	Dalit
Suicidal thoughts or acts	“I feel tense when someone blames me for not having a son; then I wish to die with the baby.”	8	Dalit
	“I have lots of *tension* because of this; sometimes I feel like consuming poison because I can’t see a way out.”	5	Mandal
Agitation or slowing of movements	“I got very angry if someone said anything to me.”	7	Muslim
	“I was very irritable so which made me angry quickly, even when they showed sympathy I shouted a lot.”	7	Muslim
***ICD-10 symptoms of Generalised Anxiety Disorder***			
Autonomic symptom	“I felt giddy, my palpitation increased.”	7	Muslim
Symptoms concerning chest and abdomen	“Sometimes I had back pain; sometimes chest pain, sometimes loin pain and sometimes tummy pain.”	8	Dalit
Symptoms concerning brain and mind	“I was lost and felt dizzy”	8	Dalit
	“I thought I might die.”	6	Mandal
General symptoms	“I have pins and needles (*jhujhuni* in Maithili; *jhamjhamaaunu* in Nepali), weakness and fear.”	10	Muslim
Symptoms of *tension*	“I had severe headache; didn’t feel like doing anything	7	Muslim
	“I had headache because of tension… I had body ache too.”	5	Dalit
Non-specific symptoms	“I get scared when someone speaks loudly.”	10	Muslim
	“I have so much *tension* so can’t remember a lot of things.”	7	Muslim
***Symptoms related to tension only***			
Disorientation	“I was wandering around here and there and got lost a few times.”	5	Sudi/Teli
Emotional pain	“One pain is for not having a son.”	8	Dalit
	“People keep blaming me for not having a son; that hurts me very much.”	8	Dalit
Self-neglect	“They were asking me why I looked tense and didn’t take care myself.”	5	Sudi/Teli
Fear for the future	“I was so apprehensive about my children!”	7	Muslim
	“I was afraid if anything happened to me and my husband because of this who would look after my two children.”	5	Sudi/Teli
Symptoms concerning the heart	“When it starts my whole body convulses and my heart is also not stable at that time.”	n/a	Dalit

Notes: ‘n/a’ indicates FGD participants who did not complete the GHQ-12; GAD generalised anxiety disorder.

**Figure 3:**
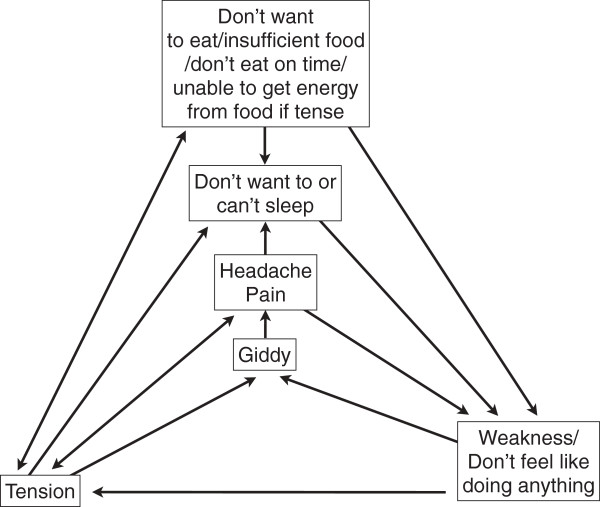
**Cycling symptoms of *tension*.** Diagram showing cycling symptoms of *tension* and weakness described by distressed mothers.

Distressed mothers defined several pathways linking affective and physical symptoms of *tension*, which were complex, rarely coherent, and involved cycling symptoms of *tension* and weakness (Figure 3). *Tension* was believed to cause physical morbidity and mortality among mothers and their infants.

Five of the distressed mothers had contemplated suicide by taking poison, though concerns about their family’s honour and who would look after their children prevented them from doing so. Suicidal thoughts were not limited to women with higher GHQ-12 scores and represented idioms of distress that even women with moderate scores would use.

*Sometimes I feel like taking poison because I can’t see a way out. […] [But] if I die, what will happen to my three children? My husband is not a responsible person. My parents will look after my children until they die, but after my parents die who will look after my children? They will become street children and people might taunt and abuse them if I take my own life.* (Amala Devi, mother, aged 24, Mandal caste, GHQ-12 score of 5, Bhindi VDC)

### Causal conditions for distress

Apprehension about the future and vulnerability gave rise to *tension*. Distressed mothers who anticipated failing to fulfil their reproductive obligations feared being discarded by their guardians and experienced *tension*. Four mothers had struggled to become pregnant and guardians and neighbours had subsequently abused them verbally and physically.

*[My husband married again] probably because I didn’t have a baby - that is what everybody was saying. […] I got married when I was a kid […] 20-25 years ago […] I was living with my husband for the last ten years but couldn’t get pregnant. Once I had a miscarriage after four months of pregnancy. I had some issues with my husband, but later we got on and I had a baby daughter. […] [My husband] does take care of me but not as much as he should. […] Now I have to live with his new wife there is no point getting worried about it.* (Pramila Devi, mother, aged 25, Dalit caste, GHQ-12 score of 11, Mansinghpatti VDC)

Eight distressed mothers had no sons, which was thought to be a cause of *tension* in all FGDs. Mothers without sons were taunted by their guardians and neighbours, who called them ‘*niputar*’. This is a derogatory term for women who ‘have never had a son in their wombs’ and are believed to be cursed. Sunita Devi and Amala Devi recalled how they felt tense and upset because family members and neighbours blamed them for not having any sons and making their husbands unhappy. These mothers experienced severe anxiety about whether their husbands would marry again since a second marriage would lower their status in the family and jeopardise their guardianship. All of the mothers without sons had considered sex-selective abortion of a female foetus, but had decided against it because of perceived health risks. Rina Devi did not follow postnatal confinement customs in the hope that this would cause the death of her newborn daughter. Somani Devi’s mother-in-law suggested drowning her newborn granddaughter because she was a financial burden on the family and forced her daughter-in-law to return to work in the fields less than a month after delivery. A subgroup of mothers without sons said their husbands had accepted that they would never have a son and suggested their wives undergo sterilisation. These mothers were less concerned about being discarded, but worried about paying for their daughters’ dowries and having no sons to care for them in their old age.

*Tension* was also caused by fear of death among mothers who experienced reproductive ill health and among those who were worried about surviving delivery and requiring a caesarean section. This fear arose when mothers were unable to afford healthcare, and because of concerns about who would look after their children in the event of their death. Many participants had taken out loans to pay for treatment, which exacerbated their *tension*.

*He didn’t listen to me and spent all the money on taari (alcohol) and other things. We need money for my treatment, medicine and delivery. I pleaded with him to save money during this pregnancy otherwise we have to take a loan or borrow money from someone. He did look after me for a year working as an ice cream seller, but now he has left me in my parents’ house. We have lots of loan on us.* (Rina Devi, mother, aged 30, Dalit caste, GHQ-12 score of 8, Khajuri Channa VDC)

### Strategies to alleviate tension

Distressed mothers favoured strategies to address the perceived causes of their *tension.* Mothers who had no sons intended to have multiple pregnancies until they had a son, and abort any female foetuses. Mothers who experienced fertility problems often sought allopathic medical treatment, including dilatation and curettage, a surgical procedure to remove tissue from the endometrium. Two mothers had also consulted a *dhami*, who identified several causes of their infertility, including being possessed by spirits or being cursed, and lacking religiosity. The *dhami* mentioned several treatments for distressed women:

*If someone has been affected by a ghost […] I will beat them with this stick and slap them once or twice* […] *I will tell [the distressed woman] to read Hanuman Chalisa, and on Tuesdays sit peacefully in a religious place. […] I will use witch tricks and give [her] cloves to eat, then she will feel peace in her heart.* (*Dhami*, Janakpur)

Mothers who perceived illness to be the cause of *tension* commonly consulted allopathic private doctors, though one had consulted an ayurvedic practitioner. Consultations with doctors were beneficial because mothers received medicine to treat their ailments and were able to use doctors to negotiate with their guardians for more food and rest.

Other strategies used by mothers to alleviate *tension* included sharing physical symptoms with guardians in order to reduce domestic workloads, negotiating a visit to their parents’ home and, in one case, challenging their guardians directly:

*I complained to my mother-in-law: because I have a daughter you don’t show any concern towards me or my daughters. My mother-in-law denied ignoring my daughter. I protested that you ask my 12 year-old daughter to go to the field and work but you do not want even the neighbours to neglect their sons. You discriminate between sons and daughters.* (Somani Devi, mother, aged 35, Dalit caste, GHQ-12 score of 5, Lagma Gathaguthi VDC)

When mothers were unable to overcome the cause of their distress they sought treatment for *tension* from doctors, who prescribed vitamins, analgesics and regular checkups. Others tried to accept their circumstances:

*Not everyone is blessed with a son and not everyone is rich in this world… All kinds of people live in this world. You have to be satisfied. Some people are very rich, somebody is blessed with a son, somebody is blessed with a daughter and somebody else is childless! When I started thinking like that then my tension disappeared.* (Somani Devi, mother, aged 35, Dalit caste, GHQ-12 score of 5, Lagma Gathaguthi)

### Intervening conditions

Most distressed mothers were unable to independently implement strategies to alleviate *tension* because they were not permitted to leave the house unaccompanied, and because guardians managed household finances and their daughter-in-laws’ domestic responsibilities. Mothers who had not fulfilled their wifely duties found it difficult to negotiate with their guardians. For example, Khabira Khatun’s father-in-law refused to pay for her treatment because he felt she was not his responsibility since she had failed to bear children. Guardians also complained about the cost of treatment, especially if their daughter-in-law had required treatment in the past.

Group members had visited the guardians of a distressed mother to ask them to give her a better diet and treat her with more kindness. Local women had organised a village meeting to help a woman who had been neglected and abused by her husband and guardians, but they said they would only intervene if they perceived the distress to be severe and the woman as deserving of help. Deservedness was judged according to the character of the woman and her ability to fulfil her duty as a wife. They felt that women who were strong, positive, patient, and did not think too much did not have *tension*. Those who were lazy, challenged their guardians’ authority, did not look after their families or care what people said about them did not deserve help. This implied that *tension* could be associated with undesirable attitudes and behaviour, and that distressed mothers may not deserve external intervention.

*My in-laws fight with me a lot. My husband will protect me but if he won’t be here I can’t do anything!* (Sunita Devi, mother, aged 25, Dalit caste, GHQ-12 score of 9, Mansinghpatti VDC)

Most distressed mothers’ husbands worked abroad, usually for several years at a time, and provided limited support through telephone conversations. These mothers described how they felt safe and relieved when their husbands returned home, especially for the birth of a child and when they were ill. Their husbands were able to help them implement strategies to alleviate *tension* by providing financial resources and negotiating with guardians on their behalf. Naima Khatun’s husband asked his parents to provide good food and care for her, and Sunita Devi’s husband intervened to prevent her being physically abused by his father:

*When my husband came here on holiday from a foreign country I didn’t let him go. I stopped him […] [My father and mother-in-law] quarrelled with me again. My father-in-law beat me in front of my husband! My husband took me to my parents’ home and called a village council meeting. […] After the meeting, when I came back from my parents’ home, nobody has said anything to me. I can lead my life the way I want! […] I have no tension now.* (Sunita Devi, mother, aged 25, Dalit caste, GHQ-12 score of 9, Mansinghpatti VDC)

Women’s group members explained that alliances between spouses threatened to disrupt household hierarchies, although opportunities for these alliances were limited since most husbands worked abroad. Beyond the marital home, distressed mothers turned to their own parents when they were unable to negotiate with their guardians and husbands for support and financial resources. Mothers described how their parents took out large loans to cover their healthcare costs.

### Consequences

Distressed mothers with successful strategies overcame their *tension* and described feeling “relieved”, “free” and “light”. Mothers whose strategies had failed to alleviate *tension*, but whose guardians and husbands continued to be supportive, said that they would continue trying to change their circumstances. For example, Saikala Khatun resolved to find treatment for her headache, abdominal and back pain and palpitations, having previously spent 20,000 Nepalese Rupees ($228) on consultations with a local doctor, a doctor in Kathmandu and a *dhami*. Rina Devi and Amala Devi had been abandoned by their husbands and forced to live with their parents. Because of social pressure for wives to live with their husbands they considered this a temporary solution and vowed to rebuild their marital relationships.

*I can’t do anything about that. I have to live with my husband’s new wife. At least I also have a baby. It doesn’t matter whether it’s a son or a daughter.* (Pramila Devi, mother aged 25, Dalit caste, GHQ-12 score of 11, Mansinghpatti VDC)

Distressed mothers who were unable to negotiate financial resources and support resigned themselves to enduring their fate. Some hoped their problems would be resolved in the future, but accepted that they could not control what happened in the interim. Some mothers had consulted extensively with doctors and *dhami*s though their symptoms persisted. This caused financial strain on families, which led to hostile relations between some mothers and their guardians, as well as additional *tension*.

## Discussion

### Interpreting tension: whose opinion matters?

We developed a model to describe psychological distress among mothers in rural Dhanusha. Participants in this and other South Asian studies used the word *tension* to express distress*. Tension* related to a cluster of mainly physical symptoms associated with social difficulties [3-5,23-25]. Fatigue and ill health may account for some of these physical symptoms, since mothers had large childcare and work burdens, and malnourishment as well as infectious and parasitic diseases are common in these communities [26,27]. Symptoms may also be somatic and used by mothers to express psychosocial distress. Evidence suggests that somatisation is a universal phenomenon, though possibly more present in collectivistic than in individualistic cultural contexts, and can be a culturally transparent and adaptive strategy to communicate distress when expressing it is disruptive to social ties [28,29].

There were no apparent differences in the experience of *tension* between Hindu and Muslim mothers. Muslim communities in Nepal are generally poorer and more marginalised than Hindu communities, and Muslim women may have more restricted mobility, potentially reducing access to social support [30]. This combination of factors is likely to put Muslim women at increased risk of *tension*, exacerbate their symptoms and affect how they express distress. However, there is inter-mixing of Muslim and Hindu communities in Dhanusha, to the extent that Hindu communities have adopted traditionally Muslim practices including *purdah,* which requires women to conceal their bodies and faces in the presence of marital relatives and men, and to avoid being seen in public by remaining in the home [31]. Furthermore, experiences of poverty, domestic abuse, absent husbands and health problems were common to both rural Muslim and Hindu mothers in our study, suggesting similar contexts and predisposing factors for *tension*.

Several perspectives influence the interpretation of what causes perinatal distress in Dhanusha, as well as recommendations on what should be done to address it. From a mental health perspective, symptoms identified in mothers’ narratives map directly onto ICD-10 diagnostic criteria for depression and anxiety, and *tension* may be considered as a somatised form of these disorders [32]. The overlap of symptoms is taken as evidence for the universal validity of ICD-10 diagnoses of depression and anxiety. However, this perspective does not acknowledge the limitations of existing psychiatric diagnostic systems: just as depression and anxiety are clusters of symptoms related to social burdens, so too is *tension*, and there is little to delineate these disorders from normal reactions to harsh living conditions [33,34]. Conceiving of *tension* as a diagnostic category, rather than an experience encompassing both a biological reality and a culturally contingent experience, reduces its meaning significantly [32]. This has implications for treatment and prevention of distress: although traditional psychiatric interventions, such as antidepressants and psychological therapies, can assist the biological and psychological consequences of mental illness, they are unlikely to address underlying social factors including poverty and gender-based victimisation.

The second perspective draws upon feminist theory and the role of gender-based victimisation in generating distress. In Nepal and other parts of South Asia, men are often accorded a higher status than women because they continue the family name, perform funeral rites and provide financial security for their parents in later life [35-37]. Women’s duties are largely reproductive and domestic [38]. They are dependent on family members to provide for and protect them, and they lack agency concerning decisions about healthcare, fertility, nutrition, their children’s education and health, and movements outside the home. They may be subject to domestic violence, verbal abuse, early marriage, polygamy and pressure to bear sons, which are known risk factors for perinatal psychological distress [11,39-41]. Although benefits of interventions to address gender-based inequities could be envisaged, mothers were concerned with being dutiful wives and did not explicitly mention the need to challenge gender inequities. It is possible that women have internalised the structural violence to which society exposes them, and cope with this by striving to be dutiful. They may be unable to imagine a situation that is more gender-balanced and therefore do not consider demanding it. However, there is a risk of imposing western ideology and perceiving women in contemporary cultures as our predecessors in a universal movement towards gender equality [42]. Furthermore, it may be over-simplistic to assume that all women in Dhanusha lack agency. An anthropological study of high caste rural Pahadi women found that they were paradoxically powerful and powerless, and that a woman’s power was in her sexuality. This power could be used positively to bear children within the patrilineage, or negatively to lure her husband away from his family [38].

Arguably the most important perspective is that of distressed mothers themselves, who mainly attributed distress to fertility problems, lack of sons and ill health. Being in debt was an additional stressor since families who could not afford healthcare were forced to take out loans. At face value, this viewpoint suggests the absence of ‘endogenous’ depression, and that distress was fundamentally reactive. However, it is possible that mothers were using a narrative of social criticism involving gender and social subordination that is itself an idiom of distress. Rather than wanting to treat *tension*, mothers wanted practical solutions to address its perceived causes, supporting a social determinants approach to intervention. However, it is a major public health concern that they wanted and seemingly had access to sex-selective abortions, and that estimates suggest the sex ratio at birth in this population is 902 female infants per 1000 male infants [10].

Integration of these three perspectives is necessary to build a more robust and meaningful evidence base, and to design and implement more culturally relevant mental health interventions. For example, screening tools could be developed that are sensitive to somatoform presentations of illness based on legitimate presentations of distress. Psychological interventions could be used to empower vulnerable women, and to support and strengthen marital relationships. Programmes could address structural violence through improved maternal and child health, increased access to healthcare, and poverty alleviation. Participatory interventions to promote gender rights could be developed through consultation with the local community and facilitated by local women. Education should be a key strategy to empower women and change gender attitudes.

### Strengths and limitations

Most of the data collection team for this study had lived in Dhanusha throughout their lives and spoke fluent Maithili and Nepali. However, transcripts were translated because researchers involved in the second analysis phase did not speak these languages. Translation-related decisions about word choices may have influenced the analysis, but our translators were familiar with the context and we were able to query their word choices and clarify meanings of passages where necessary. Excluding the *dhami*, we did not include male participants; future work could explore men’s perspectives on maternal psychological distress. There may be some limitations associated with use of the GHQ-12 as a screening tool, however selecting interview participants using a higher threshold score (≥5) enabled us to sample mothers with significant levels of psychological distress [43]. We were unable to carry out theoretical sampling as data were collected prior to analysis and development of concepts was limited by the data available. However, because of the large volume and richness of data we were largely able to avoid gaps in the theory. A further limitation was the unavoidable possibility of social desirability bias in participants’ responses, which may have caused them to conceal intimate issues.

## Conclusions

Perinatal psychological distress in Dhanusha is experienced as *tension*, involving physical symptoms related to social problems. Our findings suggest that more culturally relevant screening tools for distress are needed in this setting, as are grass-roots participatory interventions to address gender-based victimisation and structural violence.

## Abbreviations

FGD: Focus group discussion; GHQ-12: 12-item General Health Questionnaire; VDC: Village Development Committee; cRCT: Cluster-randomised controlled trial; ICD-10: 10^th^ Revision of the International Classification of Diseases; GAD: Generalised anxiety disorder.

## Competing interests

The authors declare that they have no competing interests.

## Authors’ contributions

KC, NS, MK and AP conceived and designed the study. KC and AP conducted training for interview and focus group discussion facilitators. BB, KG, MG, SJ, BT, RR, RT, AP, KC, NS and JM contributed to the design of the topic guides. KG, MG, BT, RR, RT, JM and SJ conducted interviews and FGDs. NS, BB, SJ and BS coordinated data collection. KC led the analysis and wrote the first draft of the paper. NS, BB, KG, MG, MJ, SJ, JM, RR, BS, BT, RT and AP contributed to the analysis. NS, MJ, AP, MK and DO edited the paper. DO, NS, DM, AC and BS were involved in design and management of the Dhanusha cRCT from which we sampled participants and obtained data. All authors commented on the paper and approved the final version.

## Pre-publication history

The pre-publication history for this paper can be accessed here:

http://www.biomedcentral.com/1471-244X/14/60/prepub
